# Standardized Prospective Intervention in Hospitalized Patients with Bacterial Pneumonia

**DOI:** 10.3390/jcm14248704

**Published:** 2025-12-09

**Authors:** María Rocío Fernández-Ojeda, María Dolores Galán-Azcona, Rosa Anastasia Garrido-Alfaro, María Victoria Ruiz-Romero, Antonio Fernández-Moyano, José Luis García-Garmendia

**Affiliations:** 1 Internal Medicine Department, Hospital San Juan de Dios del Aljarafe (HSJDA), 41930 Bormujos, Seville, Spainmariadolores.galan@sjd.es (M.D.G.-A.);; 2 Quality and Research Department, Hospital San Juan de Dios del Aljarafe, 41930 Bormujos, Seville, Spain; 3 Intensive Care and Emergency Department, Hospital San Juan de Dios del Aljarafe, 41930 Bormujos, Seville, Spain

**Keywords:** community-acquired pneumonia, mortality, standardized protocols, risk factors, empiric antibiotic therapy, multidisciplinary approach

## Abstract

**Background**: Community-acquired pneumonia (CAP) remains one of the leading causes of infectious mortality worldwide. Variability in diagnosis and management can significantly influence outcomes. **Objective**: To assess the association between the implementation of a standardized hospital protocol and clinical outcomes in patients hospitalized for bacterial CAP and to identify factors associated with in-hospital and 30-day mortality. **Methods**: An ambispective before–after study was conducted at Hospital San Juan de Dios del Aljarafe (Seville, Spain), including a retrospective phase (2019) and a prospective intervention period (2022–2023). The intervention consisted of a standardized clinical protocol supported by training sessions and a 9-item checklist. Adults (≥18 years) with clinically and radiologically confirmed bacterial CAP were included. Mortality, length of stay, and empirical and targeted antibiotic adequacy were compared between periods. In the prospective cohort (n = 169), mortality-associated factors were analyzed using multivariate logistic regression. **Results**: A total of 1610 patients were analyzed: 634 in the pre-intervention period and 976 during the intervention period. Hospital mortality was lower during the intervention (11.3% [95% CI 9.3–13.2] vs. 16.6%; [95% CI 13.7–19.5] *p* = 0.002) with an absolute risk difference of 5.3%, corresponding to an approximate number needed to treat (NNT) of 19. Median length of stay decreased slightly (8.1 vs. 7.9 days; *p* < 0.001). In the prospective cohort, in-hospital mortality was 7.7% and 30-day mortality 16.6%. The therapeutic effort limitation (aOR 9.10, 95% CI 1.36–121.57; *p* = 0.021) and lower SaO_2_/FiO_2_ (aOR per unit 0.98, 95% CI 0.97–0.99; *p* < 0.001) were independently associated with in-hospital mortality. The ARDS (aOR 4.29, 95% CI 1.05–19.93; *p* = 0.043), lower SaO_2_/FiO_2_ (aOR 0.99 per unit, 95% CI 0.98–1.00; *p* = 0.005), older age (aOR 1.06 per year, 95% CI 1.02–1.12; *p* = 0.005), and lower Barthel Index (aOR 0.97 per point, 95% CI 0.94–0.99; *p* < 0.001) were associated with higher 30-day mortality. **Conclusions**: Implementation of a standardized CAP protocol was associated with lower mortality and high antibiotic adequacy in the intervention cohort. While causal inference is limited by the non-contemporaneous before–after design, these findings support the integration of structured, multidisciplinary, protocol-driven strategies—together with periodic audit and feedback cycles—to strengthen CAP management in community hospital settings.

## 1. Introduction

Community-acquired pneumonia (CAP) is one of the leading causes of morbidity and mortality worldwide, particularly among adults with comorbidities or advanced age. It represents the most frequent infectious cause of death and the fourth leading cause of overall mortality globally [1,2,3]. In Spain, its annual incidence is estimated between 2 and 10 cases per 1000 inhabitants, with hospitalization rates of 20–40% among patients meeting severity criteria [1,4]. According to the National Statistics Institute, in 2020, 8767 deaths due to pneumonia were reported, accounting for 1.78% of all registered deaths in the country [4,5,6,7]. The case fatality rate varies depending on the setting—below 3% in outpatients but reaching up to 50% among hospitalized patients requiring intensive care and vasopressor support [3,6].

The severity and prognosis of CAP depend on multiple clinical factors and the adequacy of initial management. To stratify severity, international guidelines recommend validated scoring systems such as the Pneumonia Severity Index (PSI) described by Fine et al., the British Thoracic Society’s CURB-65 score, and predictive indices for severe community-acquired pneumonia requiring intensive care, including SMART-COP and SCAP [1,8,9]. These tools provide valuable information on disease progression and the likelihood of complications or death, guide decisions regarding outpatient versus inpatient management, and assist in determining the appropriate selection and duration of empirical antimicrobial therapy. Their use helps reduce hospitalization rates, healthcare costs, and adverse events associated with inpatient care [1,2,8,10].

Despite the high burden of CAP, many community hospitals continue to face substantial variability in diagnostic and therapeutic management, highlighting the need for standardized, protocol-driven approaches.

In this context, appropriate selection and timely administration of empirical antibiotic therapy remain among the most important determinants of prognosis. Recent studies, such as that by Ryu et al., have demonstrated that adherence to therapeutic guidelines reduces hospital stay and improves survival [11]. Nevertheless, the lack of unified protocols and delays in initiating antibiotic therapy continue to pose challenges, particularly in community hospitals.

Hospital San Juan de Dios del Aljarafe (HSJDA) is a community hospital serving a population of approximately 300,000 inhabitants. In 2019, 634 admissions for bacterial CAP were recorded, with a mortality rate of 16.6% (105 deaths), higher than expected for hospitals of similar characteristics.

To address this issue, and considering the high pneumonia-related mortality in the Aljarafe Health District (Seville, Spain), a prospective study was designed to identify risk factors associated with this mortality. The study also aimed to determine whether these factors could explain the observed outcomes and to identify potential areas for improvement to reduce mortality rates. Additionally, previous internal studies conducted from the Emergency Department had identified opportunities for improvement in pneumonia diagnosis and treatment. Taking both situations into account, a hospital-wide intervention was implemented based on a standardized clinical protocol and its monitoring through a checklist, with the aim of standardizing the clinical management of hospitalized CAP and examining its association with mortality outcomes.

Structured care bundles and checklist-based interventions have shown benefits in reducing clinical variability and improving adherence to guidelines in patients with pneumonia and other acute conditions, particularly in emergency and intensive care settings. However, evidence remains limited in community hospitals and general ward environments, and few studies have integrated real-time adherence monitoring with antimicrobial stewardship processes across the continuum of care. For this reason, a protocol-driven intervention based on a 9-item checklist was developed in our hospital to standardize key diagnostic and therapeutic decisions in the management of bacterial CAP.

Secondary objectives included assessing adherence to the diagnostic and therapeutic protocol in a sample of patients hospitalized for bacterial CAP and identifying risk factors associated with in-hospital and 30-day post-discharge mortality.

## 2. Materials and Methods

### 2.1. Study Design

A before–after ambispective study was conducted, including two distinct phases: a retrospective phase (patients hospitalized for bacterial community-acquired pneumonia [CAP] during 2019) and a prospective phase (patients admitted between January 2022 and June 2023) following the implementation of a standardized hospital intervention.

Our team began implementing the protocol in January 2022 as a pilot in order to identify potential improvements, and we found it necessary to expand the checklist and the variables to be collected prospectively. For this reason, we updated the protocol and resubmitted it to the Ethics Committee for evaluation. After reviewing it, the Committee requested some changes regarding the sample size and an extension of the statistical analysis. We made those changes and resubmitted it, and the final approval was issued by the middle of the year.

The years 2020 and 2021 were excluded due to the impact of the SARS-CoV-2 pandemic, which substantially altered the epidemiology, clinical management, and outcomes of pneumonia cases.

### 2.2. Study Population

All patients aged ≥18 years admitted to HSJDA between 1 January 2022, and 3 June 2023, with a diagnosis of community-acquired bacterial pneumonia were eligible for inclusion. Diagnosis required clinical criteria (respiratory symptoms and fever), radiological evidence (new pulmonary infiltrate), and the performance of microbiological testing (e.g., sputum or tracheal aspirate culture, urinary antigen test, or blood culture), although identification of a specific pathogen was not required for inclusion. The cases with documented viral infection or high suspicion of non-bacterial etiology were excluded.

Exclusion criteria were viral, aspiration, nosocomial, or ventilator-associated pneumonia; severe immunosuppression; active SARS-CoV-2 infection; refusal to participate in the prospective phase; or incomplete clinical data.

### 2.3. Intervention

The hospital intervention consisted of the implementation of a standardized clinical protocol for the diagnosis, treatment, and follow-up of patients hospitalized with bacterial CAP. The protocol was developed based on the 2020 guidelines of the Spanish Society of Pulmonology and Thoracic Surgery (*Sociedad Española de Neumología y Cirugía Torácica*, SEPAR) (Madrid, Spain) [12] and adapted to the hospital’s local epidemiological context.

The protocol was disseminated among all healthcare professionals involved in the management of these patients (emergency physicians and internal medicine staff) through educational sessions, printed guidelines, and supplementary training materials.

**Figure 1:**
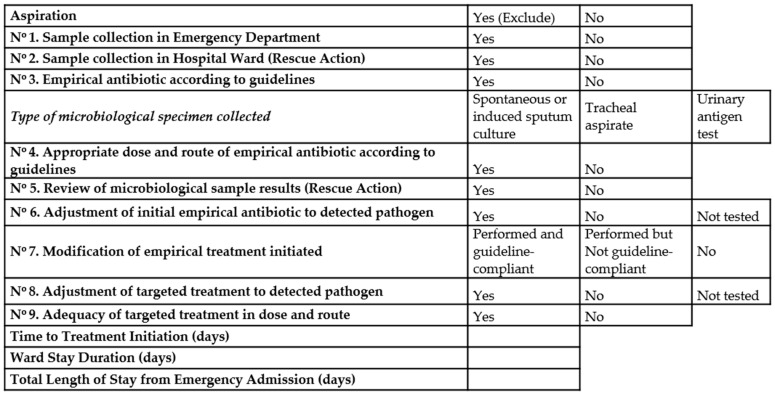
Protocol.

A checklist (Figure 1) was designed to monitor adherence and confirm protocol implementation. It included 9 key actions (7 primary and 2 rescue measures). Primary actions reflected the essential steps expected at initial evaluation, whereas rescue actions are the interventions to accomplish an optimal management of pneumonia patients. So, we consider that a higher number of rescue actions (correction of antibiotic therapy election or doses, microbiological review, new microbiological samples…) would increase the quality of management, so the potential of benefits in mortality would increase.

### 2.4. Protocol of Pneumonia Management Included

1. Diagnose of pneumonia at emergency room: Register of comorbidities and risk factors at hospital admission. Register of clinical variables, laboratory tests (biochemical, blood count, gasometry values and inflammation markers) and presence of type pulmonary infiltrates in chest x-ray. Clinical diagnose of pneumonia was based on established criteria. Microbiological specimen’s collection: blood cultures, spontaneous or induced sputum cultures, tracheal aspirate when indicated, and urinary test antigens.
2. When pneumonia diagnose was established and appropriate cultures were obtained, antimicrobial therapy should be initiated following the therapeutic guidelines of the hospital.
3. When the patients were admitted to hospital wards, the intervention group developed the survey of different items considered as important for the appropriate management and described in Figure 1.

In addition, a standardized data collection form was used to prospectively record all study variables for hospitalized patients during the study period. (Variables collected are detailed in the corresponding results tables).

### 2.5. Sample Size Calculation

Sample size was calculated using the GRANMO online calculator (Barcelona, Spain). Based on the retrospective cohort (2019) mortality rate of 16.6%, we assumed an expected reduction in mortality to 11.0% (absolute risk reduction 5.6 percentage points, relative risk ≈ 0.66), with a 95% confidence level (α = 0.05) and 80% power (β = 0.20). A sample of 466 patients was required in the pre-intervention group and 932 in the intervention group, assuming a 5% loss rate. The arcsine approximation for a two-tailed comparison of proportions was applied.

### 2.6. Data Collection

**Figure 2:**
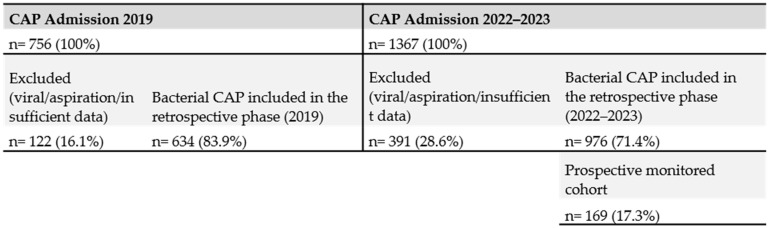
Study flow chart (STROBE).

During the period following the intervention (2022–first half of 2023), a total of 1367 hospital admissions with a diagnosis of community-acquired pneumonia (CAP) were recorded. After excluding viral and aspiration pneumonias, approximately 976 cases remained. Among them, 162 were coded as bacterial pneumonia and 814 as “pneumonia due to unspecified microorganism.” Although most of these latter cases were likely bacterial in origin, the absence of microbiological testing prevented their classification as confirmed bacterial CAP (Figure 2).

Of these 976 eligible patients, a prospective subset of 169 consecutive cases was enrolled and managed according to the new standardized protocol. This subset included all patients in whom complete monitoring using the standardized checklist and comprehensive variable recording were feasible. The selection of this cohort was pragmatic and based on the availability of human and logistical resources, as it was not possible to prospectively monitor all admissions. The expectation was that the procedural changes and standardized practices introduced through the intervention would progressively extend to all hospitalized CAP patients, which was confirmed during the study period. The prospective cohort of 169 patients was not powered for multivariable mortality modeling; therefore, these analyses should be interpreted as exploratory (Figure 2).

All predefined study variables were collected to evaluate both (1) adherence to the clinical protocol and (2) factors associated with mortality among patients admitted with community-acquired pneumonia. Consecutive sampling was used, including all eligible patients admitted during the study period.

The retrospective group (2019) served as a control, representing the last pre-pandemic year with stable and representative data. The following variables were recorded: demographic, clinical, laboratory, and radiological parameters; key prognostic factors (age, comorbidities, Barthel Index (Maryland State Medical Society, Baltimore, MD, USA), CURB-65 score (University of Nottingham, Nottingham, UK), oxygenation ratio [SaO_2_/FiO_2_], laboratory findings, isolated pathogen, antibiotic appropriateness, complications during hospitalization, and mortality [in-hospital and 30-day post-discharge]). The degree of protocol adherence (number of checklist items completed) and rescue actions performed were also quantified.

The FiO_2_ value was estimated for non-ventilated patients according to standard conversion tables: nasal cannula 1–6 L/min = FiO_2_ 0.24–0.44, Venturi mask according to manufacturer fraction, and reservoir mask up to FiO_2_ 0.80–0.90. For high-flow nasal oxygen and mechanical ventilation, FiO_2_ corresponded to device settings. This approach has been used and validated in previous studies of hypoxemia assessment in pneumonia and acute respiratory failure [13].

30-day mortality was defined as death occurring within 30 days from the date of hospital admission. Post-discharge deaths were identified through the integrated electronic health record of the Andalusian Public Health Service (Seville, Spain), which links hospital, primary care, and regional mortality registry data. This system allows confirmation of deaths occurring at home or in other hospitals within the public network. Patients transferred to other hospitals remained traceable through the same regional database, ensuring complete follow-up for the 30-day outcome.

Data sources included the electronic medical record, the laboratory database, and the hospital pharmacy database. Patient complexity and mortality risk were stratified using the Diagnosis-Related Groups (DRG) classification system, employed by the Spanish National Health System (Madrid, Spain) to categorize patients according to clinical severity and hospital resource utilization.

### 2.7. Statistical Analysis

Statistical analyses were performed using IBM SPSS Statistics (IBM Corp., Armonk, NY, USA), version 27.0. Quantitative variables were expressed as mean ± standard deviation or median (interquartile range, Q1–Q3) according to distribution. Qualitative variables were expressed as frequencies and percentages.

Missing data were not imputed; all analyses were performed using complete-case analysis.

For independent variable comparisons, Student’s *t*-test and ANOVA were used for normally distributed quantitative variables, and the Mann–Whitney *U* and Kruskal–Wallis tests for non-parametric distributions. Qualitative variables were compared using the Chi-square or Fisher’s exact test (for low expected frequencies).

### 2.8. Additional Pre/Post Risk-Adjusted Analysis

To further explore the association between the implementation of the standardized protocol and in-hospital mortality, we performed an additional multivariable logistic regression on the combined 2019 and 2022–2023 cohorts (n = 1610). The dependent variable was in-hospital death, and the main exposure was a binary indicator for the post-implementation period (2022–2023 vs. 2019). The model was adjusted for age (continuous), sex, and DRG mortality risk category (mild, moderate, high, extreme), which were the only patient-level covariates available and consistently defined across both periods. Clinical severity variables such as CURB–65, SaO_2_/FiO_2_ and laboratory parameters were only available for the prospective 2022–2023 cohort and were therefore not included in the combined pre/post model.

### 2.9. Multivariable Analyses in the Prospective Cohort

In the prospective cohort, multivariable modelling was performed using Firth’s penalized likelihood logistic regression (logistf package in R (R Project version 4.4.1.), R Foundation for Statistical Computing, Vienna, Austria), given the limited number of deaths and evidence of quasi-separation in standard logistic models. For in-hospital mortality, we specified a parsimonious model including sex, guideline-concordant empirical antibiotic therapy, therapeutic effort limitation, SaO_2_/FiO_2_ ratio, and age. For 30-day post-discharge mortality, the model included ARDS, rescue actions, septic shock, SaO_2_/FiO_2_ ratio, age, and Barthel Index at admission.

These penalized models were constructed with an “etiologic aim” (to identify factors associated with mortality) rather than as formal prognostic scores. The set of candidate predictors was defined a priori based on clinical relevance and prior evidence (e.g., age, functional status, ARDS, shock, oxygenation, guideline-concordant antibiotics, therapeutic effort limitation) and the initial multivariable analyses (which were intentionally overinclusive and revealed which variables were consistently associated with mortality). For the in-hospital mortality* model, we retained a very small, prespecified set of five predictors: sex, guideline-concordant empirical antibiotic therapy, therapeutic effort limitation, SaO_2_/FiO_2_, and age. For the 30-day mortality model, we prespecified ARDS, rescue actions, septic shock, SaO_2_/FiO_2_, age and Barthel Index. We did not use automated stepwise selection; instead, we deliberately limited the models to a handful of clinically justified variables to reduce overfitting in line with the reviewer’s recommendations. The multivariable models were fitted on complete cases for the included covariates because the proportion of missing data for these predictors was low.

We checked for potential collinearity among candidate predictors. To do this, we calculated variance inflation factors (VIF) using the *vif* function from the rms package in R (R Project version 4.4.1.). The VIF values well below commonly used thresholds for concern (e.g., VIF > 5–10). These results, together with the bivariate correlations, suggest that collinearity among predictors is not a relevant issue in our penalized models.

Nevertheless, we assessed internal discrimination and calibration to quantify overfitting. For each endpoint, we performed bootstrap internal validation, refitting the penalized model in multiple resamples and evaluating the area under the ROC curve (AUC) and calibration intercept and slope both in the bootstrap and in the original samples. The average optimism was subtracted from the apparent AUC, intercept and slope to obtain optimism-corrected estimates. We report Firth-penalized adjusted odds ratios (aORs) with 95% confidence intervals, together with apparent and optimism-corrected AUCs and calibration intercepts and slopes.

## 3. Results

In the pre-intervention group (2019), a total of 756 patients were hospitalized with community-acquired pneumonia (CAP) at HSJDA, of whom 634 cases (83.9%) were of bacterial etiology. In the intervention period (2022 to the first half of 2023), 1367 patients were admitted for CAP, with 976 (71.4%) confirmed as bacterial.

Considering the reference population of approximately 300,000 inhabitants served by the HSJDA healthcare district, the annual incidence of community-acquired bacterial pneumonia requiring hospitalization was 216 cases per 100,000 inhabitants in 2019 and 324 cases per 100,000 inhabitants in 2023. This increase reflects a higher absolute number of hospital admissions for bacterial CAP after the COVID-19 pandemic, in a context of population aging and possibly increased infectious susceptibility.

Age and sex distribution were similar between the two groups, although there was a slightly lower proportion of men in the intervention group (54.7% vs. 60.6%; *p* = 0.020). The latter cohort also showed higher clinical complexity, with a greater proportion of patients classified as “high” or “extreme” severity and mortality risk according to the Diagnosis-Related Groups (DRG) system used in Spanish hospitals to stratify patients by clinical severity and resource consumption (*p* < 0.001).

Following implementation of the standardized hospital protocol, a lower in-hospital mortality rate was observed (16.6% vs. 11.3%; *p* = 0.002). Median length of stay was also reduced, from 8.13 days (8.01–9.27) to 7.89 days (7.47–8.85) (*p* < 0.001). Although the reduction in median length of stay reached statistical significance due to the large sample size, the absolute difference (0.24 days) is clinically modest. Likewise, the proportion of patients requiring intensive care unit (ICU) admission increased from 2.1% to 3.8% during the intervention period. Although overall healthcare resource consumption was slightly higher in the intervention group, the difference did not reach statistical significance (Table 1). These findings suggest an improvement in clinical outcomes and healthcare efficiency, although causality cannot be inferred due to the study design.

In the risk-adjusted multivariablre logistic regression including all 1610 patients from 2019 and 2022–2023, admission during the post-implementation period remained independently associated with lower in-hospital mortality. After adjustment for age, sex, and DRG mortality-risk category, the adjusted odds ratio (aOR) for in-hospital death in 2022–2023 versus 2019 was 0.62 (95% CI 0.45–0.85; *p* = 0.003). The age was associated with higher mortality (aOR per year increase 1.07; 95% CI 1.05–1.09; *p* < 0.001), whereas sex and DRG mortality-risk strata were not significant predictors in the adjusted model.

### 3.1. Prospective Cohort Analysis (2022–2023)

A total of 169 patients were prospectively followed using the protocol adherence checklist. The median age was 77 years (IQR 63–85), and 53.3% were male. The most frequent comorbidities were chronic heart failure (41.4%), diabetes mellitus (27.2%), active smoking (21.9%), previous neurological deficit (21.3%), and chronic obstructive pulmonary disease (19.5%). Over one-third of patients (37.9%) had baseline functional dependence (Barthel Index < 100), and 26.6% had been hospitalized within the previous 12 months. The median number of comorbidities was 2.0 (1.0–3.0). Median baseline oxygen saturation (SaO_2_) was 92.0% (88.0–97.0), Barthel Index 100 (60.0–100), and CURB-65 score 2.0 (2.0–2.0). This narrow IQR reflects the homogeneous severity profile of the prospective cohort, in which most patients presented with a CURB-65 score of 2.

Laboratory and clinical parameters on admission are detailed in Table 2.

At initial emergency department assessment, 20.2% of patients presented with normal pulmonary auscultation, and the most frequent radiological finding was alveolar infiltrate (82.8%). More than half of the patients required low-flow oxygen support via nasal cannula (58.7%), and 10.8% required high-flow or mechanical ventilation support (including high-flow nasal oxygen, non-invasive ventilation, or invasive mechanical ventilation). Additionally, 68.6% received systemic corticosteroids, and 55.6% had documented pneumococcal vaccination.

Microbiological testing was performed in almost all patients (95.2%). A causative pathogen was identified in 59 of the 169 cases (34.9%), while 110 patients (65.1%) had negative or non-diagnostic results. Only six patients (3.6%) did not have microbiological samples obtained.

The most common in-hospital complications were acute respiratory distress syndrome (ARDS) in 36.7% of cases, congestive heart failure in 42.0%, acute kidney injury in 23.1%, and delirium or altered mental status in 18.9%.

Regarding outcomes, there were 13 deaths during the acute episode (7.7%) and a total of 28 deaths (16.6%) when including in-hospital and 30-day post-discharge mortality (Table 3).

Protocol adherence was high. The median number of actions completed was 5 (IQR 4–7), including four primary and one rescue action (collection of samples in the ward and review of microbiological results). Microbiological samples were obtained in the emergency department in 78.1% of cases, and 63.3% of patients received empiric antibiotic therapy consistent with hospital guidelines. Among patients with an identified pathogen (n = 64), targeted therapy was appropriate in 81.3%. The median length of stay in the prospective cohort was 5.9 days (IQR 3.7–8.4) (Table 4).

### 3.2. Factors Associated with Mortality

Bivariate analysis showed that in-hospital mortality was significantly associated with chronic heart failure (69.2% vs. 39.0%; *p* = 0.038), ICU admission (23.1% vs. 3.8%; *p* = 0.023), need for invasive mechanical ventilation (*p* < 0.001), and ARDS development (*p* < 0.001). Other significant associations included congestive heart failure (*p* = 0.038), altered mental status (*p* = 0.019), and a formal limitation of therapeutic effort (*p* < 0.001) (Table 5).

Among variables associated with 30-day post-discharge mortality, patients who died were significantly older [84.0 (74.9–87.7) vs. 74.9 (58.7–83.7); *p* = 0.002], had a lower Barthel Index [50.0 (40.0–95.0) vs. 100 (70.0–100); *p* < 0.001], greater pre-existing dependence (67.9% vs. 31.9%; *p* < 0.001), were more frequently institutionalized (25% vs. 5%; *p* = 0.003) and had chronic heart failure (71.4% vs. 36.0%; *p* = 0.005) (Table 6).

Other factors significantly associated with 30-day mortality included hypoalbuminemia [2.40 (2.05–2.70) vs. 2.70 (2.28–2.93); *p* = 0.046], hyperuricemia [86.5 (52.5–153.8) vs. 54.0 (38.0–77.3); *p* = 0.001], lower oxygenation ratio (SaO_2_/FiO_2_) [201.9 (SD 94.70) vs. 323.2 (SD 87.81); *p* = 0.002], ARDS (78.6% vs. 28.4%; *p* < 0.001), acute kidney injury (50.0% vs. 17.7%; *p* < 0.001), septic shock (28.6% vs. 7.1%; *p* = 0.003), extrapulmonary infection (28.6% vs. 12.1%; *p* = 0.038), congestive heart failure (67.9% vs. 36.9%; *p* = 0.002), and myocardial ischemia (14.3% vs. 2.1%; *p* = 0.015). Notably, a lower proportion of microbiological sampling in the emergency department was observed among patients who died within 30 days after discharge (64.3% vs. 81.4%; *p* = 0.044) (Table 6).

### 3.3. Multivariate Analysis. Prospective Cohort

In the Firth-penalized model for in-hospital mortality (13/169 deaths), therapeutic effort limitation (aOR 9.10, 95% CI 1.36–121.57; *p* = 0.021) and lower SaO_2_/FiO_2_ (aOR per unit 0.98, 95% CI 0.97–0.99; *p* < 0.001) were independently associated with death, whereas sex, age and guideline-concordant empirical antibiotic therapy were not statistically significant. The model showed high apparent discrimination with an apparent area under the ROC curve (AUC) of 0.96, with an optimism-corrected AUC of 0.94; the optimism-corrected calibration intercept and slope were −0.18 and 0.37, respectively, indicating substantial overfitting and reinforcing the exploratory nature of this analysis (Table 7).

In the Firth-penalized model for 30-day mortality (28/168 deaths), ARDS (aOR 4.29, 95% CI 1.05–19.93; *p* = 0.043), lower SaO_2_/FiO_2_ (aOR 0.99 per unit, 95% CI 0.98–1.00; *p* = 0.005), older age (aOR 1.06 per year, 95% CI 1.02–1.12; *p* = 0.005), and lower Barthel Index (aOR 0.97 per point, 95% CI 0.94–0.99; *p* < 0.001) were associated with higher 30-day mortality, while rescue actions and septic shock did not remain significant after penalization. Apparent discrimination was again high (AUC 0.94), with an optimism-corrected AUC of 0.91 and an optimism-corrected calibration intercept and slope of −0.18 and 0.83, respectively (Table 7).

### 3.4. Trend Analysis of Standardized Mortality Rates

**Figure 3:**
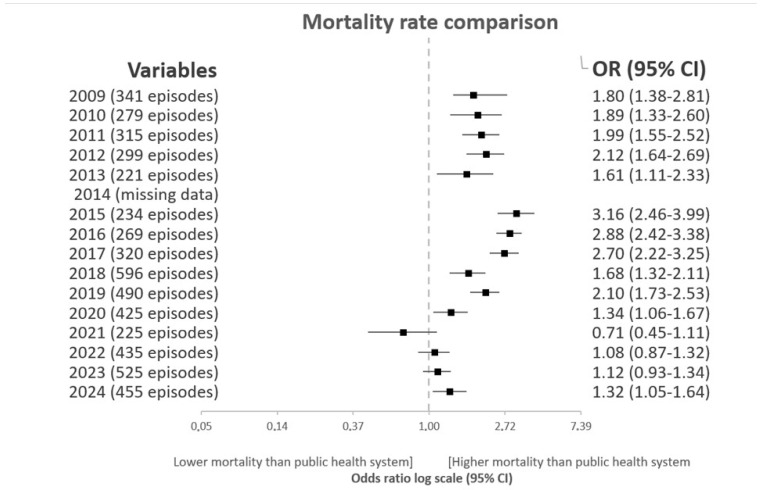
Mortality rate comparison Hospital San Juan de Dios del Aljarafe with the whole andalusian public health system rates (Agency for Healthcare Research and Quality).

Figure 3 shows the standardized pneumonia mortality rates at HSJDA compared with those of the Andalusian public healthcare system, analyzed using the Agency for Healthcare Research and Quality (AHRQ) methodology. Between 2009 and 2019, pneumonia mortality at HSJDA consistently exceeded the Andalusian average, with adjusted odds ratios ranging from 1.6 to 3.2. However, a progressive decline has been observed since 2021, reaching values comparable to the regional average in 2022 (OR 1.08; 95% CI: 0.87–1.32) and 2023 (OR 1.12; 95% CI: 0.93–1.34). This downward trend coincides temporally with the implementation of the standardized hospital protocol and may reflect improved clinical outcomes and alignment with regional quality standards.

During the year 2024, when the intervention was no longer implemented, we observed a significant increase in the pneumonia mortality rate, once again rising above the expected average. In response to this situation, measures have been initiated to improve adherence to the protocols, including the consideration of incorporating artificial intelligence tools.

## 4. Discussion

This study demonstrates that the implementation of a standardized hospital protocol for the management of community-acquired pneumonia (CAP) was associated with lower mortality and better clinical outcomes at the HSJDA. Standardizing the clinical care process—supported by professional training and compliance monitoring—appeared to promote greater consistency in clinical practice and was associated with high antibiotic stewardship performance in the intervention period and healthcare efficiency, even in a context of increased clinical complexity according to Diagnosis-Related Group (DRG) classification [14].

The main finding was a significant decrease in in-hospital mortality, from 16.6% in the pre-intervention group to 11.3% after protocol implementation, representing a 32% relative reduction. This result support the potential benefit of structured and coordinated approaches to CAP management, consistent with previous evidence supporting protocol-driven and multidisciplinary strategies to improve outcomes in hospitalized patients [15,16,17,18]. Nevertheless, mortality remains high, reflecting the intrinsic severity of CAP, particularly among older adults and those with significant comorbidities [6,19].

To strengthen the interpretation of these findings, we conducted an additional risk-adjusted analysis combining the pre-intervention and intervention cohorts. Even with the limited covariates available for both periods (age, sex, and DRG mortality-risk category), admission during 2022–2023 remained independently associated with lower in-hospital mortality. Although this supports the robustness of the association, it does not eliminate the possibility that unmeasured confounding or differences in patient profiles between periods could have contributed to the observed effect.

Despite the temporal association between the intervention and the observed improvement in outcomes, the before–after design does not allow causal inference. Differences in patient characteristics, hospital activity, and the post-pandemic clinical context could also have influenced the results.

A limitation of this study is the small number of events, 13 in-hospital deaths and 5 predictors in the Firth-penalized model and 28 deaths within 30 days of discharge and 7 predictors (plus intercept). We acknowledge that these ratios are below conventional heuristics (e.g., 10 events per variable). This is precisely why we abandoned the original, larger models, adopted Firth’s penalized likelihood, restricted the number of covariates to a small, clinically prespecified set, and performed bootstrap internal validation with optimism correction. We stress that, despite these precautions, the models remain susceptible to overfitting due to the limited number of events. The reported AUCs and calibration intercepts and slopes therefore serve primarily to document internal consistency and the degree of overfitting, not to validate a prognostic tool for routine use. As such, the multivariable findings should be regarded as exploratory and hypothesis-generating.

Furthermore, all observed differences between study periods should be interpreted as associations rather than causal effects, given the non-contemporaneous before–after design. Residual confounding and unmeasured differences in patient management or case mix between 2019 and 2022–2023 cannot be excluded. Future controlled or multicenter studies would be needed to confirm these findings and to better delineate the contribution of standardized care protocols to patient outcomes.

In addition, relevant secular trends may have influenced outcomes independently of the intervention. The intervention cohort showed a higher proportion of patients classified as high or extreme severity according to DRG categories, reflecting a shift toward a more complex case mix after the COVID-19 pandemic. Such differences in baseline severity, together with broader post-pandemic changes in epidemiology, vaccination coverage, healthcare utilization and coding practices, may partly account for variations observed between the two periods. These factors further limit causal interpretation despite the temporal association with the intervention.

Mortality trends observed in this study also align with the regional standardized rates. As shown in Figure 1, between 2009 and 2019 pneumonia-related mortality at HSJDA consistently exceeded the average of the Andalusian public health system, with adjusted odds ratios above 1.5. However, a progressive decline has been observed since 2021, reaching rates in 2022 and 2023 that were nearly equivalent to regional benchmarks. This convergence suggests a possible relationship between the intervention and improved quality indicators.

Another relevant observation was the apparent increase in the incidence of hospitalized bacterial CAP between 2019 and 2023, from 216 to 324 cases per 100,000 inhabitants. This rise may reflect both improved case identification and recording after the intervention, as well as a genuine post-pandemic increase in respiratory infections, driven by population aging and waning community immunity. Recent studies from different settings support these trends [5,20,21,22].

It is also important to consider the potential influence of secular post-COVID trends when interpreting these results. Several systemic changes—including shifts in respiratory epidemiology, vaccination coverage, hospital workflows, coding practices, and staffing patterns—could have contributed independently to outcome differences between 2019 and 2022–2023. Moreover, patients admitted in the intervention period presented higher DRG-based severity and mortality-risk categories, indicating a more complex case mix. These temporal and demographic changes may partly account for improvements in outcomes and reinforce the need for cautious interpretation of the findings.

Analysis of risk factors associated with mortality identified key variables that should be considered in the clinical assessment of CAP patients. Advanced age, chronic heart failure, functional dependence, and low oxygen saturation at admission were associated with poorer outcomes [19,23,24,25,26], whereas the appropriate guideline-concordant empirical therapy—frequent in the intervention cohort— was associated with a significant reduction in mortality risk [1,3,27]. These findings align with prior research highlighting the combined effect of comorbidities, functional impairment, and altered clinical parameters—such as temperature and hypoxemia—as major prognostic determinants in CAP. The observed association between ventilatory support requirements, the development of acute respiratory distress syndrome (ARDS), and mortality is also consistent with previously described severity predictors in CAP [1].

An additional notable finding was the association between “limitation of therapeutic effort” and higher in-hospital mortality. This suggests that a substantial proportion of patients with severe CAP present baseline conditions that warrant treatment-limiting decisions, underscoring the need for an integrated approach that includes both curative and palliative dimensions of care. Incorporating advance care planning and comprehensive geriatric assessment could help optimize management in this subgroup of patients [28,29,30,31]. These findings also raise important ethical considerations. Treatment-limiting decisions should be based on individualized assessments of prognosis, patient preferences, and quality of life, ideally through shared decision-making and advance care planning. Integrating palliative care principles in this context can help align therapeutic goals with patient values and avoid non-beneficial interventions.

Adherence to the hospital protocol demonstrated a clinically meaningful impact, particularly when using structured checklists ensuring high rates of appropriate initial and targeted antibiotic therapy in the intervention cohort. This highlights the importance of structured interventions to guarantee adherence to best evidence-based practices [1,2,10]. Evidence supports that protocol-based interventions, including systematic culture review and therapy adjustment according to microbiological results, enable more precise and rational antimicrobial use—an essential aspect in the context of increasing bacterial resistance and selective antibiotic pressure [1,2,32].

However, a limitation of the study is that we do not have data from the prospective cohort to assess the appropriateness of the prescribed antibiotic and adherence to guidelines, which would allow us to compare it with the appropriateness of the intervention. For this reason, we can only state that there was high appropriateness in the intervention simple.

The association observed between performing two rescue actions and higher 30-day mortality is not interpreted as a causal effect of the protocol. Instead, it likely reflects residual confounding by severity: patients requiring additional corrective steps (e.g., repeated microbiological sampling or therapy adjustments) are typically those with more severe presentations or unexpected clinical deterioration. Therefore, the number of rescue actions should be considered a marker of underlying complexity rather than an independent determinant of prognosis.

Although the before–after design precludes causal inference, several mechanisms support the plausibility of this association. Protocol adherence ensures timely cultures, appropriate empirical therapy, and targeted antimicrobial optimization, which are known to reduce treatment delays, decrease inappropriate antibiotic exposure, and improve clinical stability in CAP. These mechanisms have been consistently associated with improved prognosis in prior studies and may explain, at least in part, the observed outcome differences.

Moreover, during the year 2024, after the intervention had ended, mortality again deviated above the average, once more exceeding the expected rate. Following this finding, measures have been initiated to improve adherence to the protocols, including the consideration of incorporating artificial intelligence tools.

Standardization of care processes through local clinical protocols and educational initiatives contributes to reduced mortality, shorter hospital stays, and decreased clinical variability, without increasing resource consumption [33,34,35]. Antimicrobial stewardship programs have demonstrated that adherence to best practices, systematic monitoring of compliance, and transition to oral therapy based on clinical stability criteria improve patient outcomes and healthcare efficiency [1,2,3,33]. The American Thoracic Society and the Infectious Diseases Society of America recommend individualized antibiotic therapy based on disease severity, risk factors, and local epidemiology [1,2,3,35].

In this study, favorable effects were observed despite greater patient complexity and severity, reinforcing the efficacy of the implemented intervention. The effectiveness of structured quality-improvement strategies for CAP lies in clear protocols, continuous staff training, and systematic monitoring of adherence, leading to improved guideline compliance, optimized antibiotic use, reduced mortality, shorter hospital stays, and more efficient resource utilization, without increasing healthcare costs [31,36,37,38]. Although our study was not designed to compare pre-intervention and intervention antibiotic adequacy, we did observe high rates of guideline-concordant empirical therapy in the intervention cohort. Multifaceted interventions such as care bundles, audit and feedback, and medical education have been shown to enhance clinical outcomes and organizational performance, and are recommended by the American Thoracic Society and the Infectious Diseases Society of America [39]. This model is both applicable and transferable to hospitals seeking to standardize care and optimize outcomes in this prevalent disease [38,40,41].

The ambispective design of the present study, including a retrospective pre-intervention cohort, was justified by the need to use data from before the COVID-19 pandemic, which profoundly altered the epidemiology and management of pneumonia. A concurrent non-intervention group could not be included, as the protocol was implemented hospital-wide. These features represent study limitations, along with its single-center nature, which may restrict the generalizability of the findings. However, the large sample size, the quality of data collection, and the internal consistency of results strengthen the validity of the conclusions.

Additionally, the absence of a concurrent control group limits internal validity and increases the likelihood that secular post-COVID trends or unrelated organizational or epidemiological changes may partially explain some of the differences observed between study periods. Therefore, outcome comparisons should be interpreted with caution.

A further limitation relates to the size and selection of the prospective cohort. The inclusion of 169 patients reflected a pragmatic decision driven by the availability of human and logistical resources, as it was not feasible to prospectively monitor all admissions. Although only part of the eligible population could be followed with full checklist monitoring, the standardized procedures introduced during the intervention were progressively adopted across all hospitalized CAP patients during the study period, reducing the risk of systematic selection bias. In addition, during 2022–2023, 976 cases met inclusion criteria after excluding viral and aspiration pneumonias; however, only 162 were coded as bacterial pneumonia, while 814 were coded as ‘pneumonia due to unspecified microorganism,’ largely due to the absence of microbiological testing, which prevents classification despite likely bacterial etiology. These contextual factors explain the numerical difference between the total eligible population and the prospectively monitored cohort. Other limitation was that the time to the first antibiotic dose was collected in days rather than hours due to limitations of the electronic health record. Therefore, a more detailed analysis of the delays is not possible.

Future research should aim to expand this analysis using multicenter and controlled designs to confirm external validity and explore other relevant outcomes, such as readmission rates, post-discharge quality of life, or the economic impact of the intervention. Longer follow-up could also assess the sustainability of the protocol and its influence on antimicrobial resistance. Integrating digital clinical decision-support tools and automated audit systems would represent a further step toward continuous quality improvement.

## 5. Conclusions

In conclusion, the implementation of a standardized protocol for the diagnosis and management of CAP at HSJDA was associated with lower mortality and indicators of improved quality of care. However, given the before–after design, these findings should be interpreted as associations rather than causal effects.

Mortality improvements persisted after limited risk adjustment; however, causal inference is not possible, and residual confounding and secular post-pandemic changes cannot be excluded.

Despite these positive results, mortality remains high in this high-risk population, emphasizing the need for a multidisciplinary approach integrating preventive, therapeutic, and palliative strategies, alongside sustained programs for continuous clinical improvement and practice evaluation.

To ensure long-term sustainability, future implementation should incorporate periodic audit and feedback cycles, ongoing staff training, and continuous monitoring of adherence and clinical outcomes.

## Figures and Tables

**Table 1:** Comparison of Mortality from Community-Acquired Bacterial Pneumonia Before and During the Intervention.

	Before (2019) N (%)	After (2022–2023) N (%)	Significance
Patients with community-acquired bacterial pneumonia	634 (39.4)	976 (60.6)	
Sex (male)	384 (60.6)	534 (54.7)	0.020 *
Severity level			<0.001 *
Mild	141 (22.2)	142 (14.5)	
Moderate	301 (47.5)	430 (44.1)	
High	160 (25.2)	322 (33.0)	
Extreme	32 (5.0)	82 (8.4)	
Mortality risk			<0.001 *
Mild	112 (17.7)	120 (12.3)	
Moderate	321 (50.6)	314 (32.2)	
High	160 (25.2)	388 (39.8)	
Extreme	41 (6.5)	154 (15.8)	
ICU ^1^ admission	13 (2.1)	37 (3.8)	0.049 *
Discharge due to death	105 (16.6) (95% CI 13.7–19.5)	110 (11.3) (95% CI 9.3–13.2)	0.002 *
	**Median (Q1–Q3) ^2^**	**Median (Q1–Q3) ^2^**	**Significance**
Age (years)	79.1 (67.8–86.5)	78.5 (66.5–86.2)	0.455
Resource intensity weight	0.605 (0.605–0.956)	0.711 (0.583–0.850)	0.061
Average length of stay (days)	8.13 (8.01–9.27)	7.89 (7.47–8.85)	<0.001 *
	**Multivariate Coefficient (B)**	**Multivariate OR ^3^ (95% CI) ^4^**	**Significance**
Period (2022–2023)	0.48	0.62 (0.45–0.85)	0.003 *
Age (years)	0.07	1.07 (1.05–1.09)	<0.001 *
Sex (female)	0.21	0.81 (0.60–1.10)	0.172
Mortality risk (moderate)	0.36	1.44 (0.87–2.37)	0.153
Mortality risk (high)	0.48	1.62 (0.97–2.71)	0.066
Mortality risk (extreme)	0.36	1.43 (0.77–2.69)	0.261

* Statistical significance *p* < 0.05. ^1^ ICU: intensive care unit; ^2^ Q1: Quartile 1; Q3: Quartile 3. ^3^ OR: Odds Ratio; ^4^ 95% CI: 95% confidence interval.

**Table 2:** Sample Characteristics at Admission.

Sample Characteristics	N (%)
**Gender**	
Male	90 (53.3)
Female	79 (46.7)
**Comorbidities**	
Active smoking	37 (21.9)
Active alcohol use	30 (17.8)
Use of other drugs	8 (4.7)
Immunosuppressed	29 (17.2)
Active neoplastic disease	18 (10.7)
Chronic heart failure	70 (41.4)
Chronic liver failure	10 (5.9)
Chronic kidney failure	33 (19.5)
Pre-existing neurological deficit	36 (21.3)
Chronic obstructive pulmonary disease	33 (19.5)
Diabetes mellitus	46 (27.2)
Pre-existing dysphagia	8 (4.7)
**Other Possible Risk Factors at Admission**	
Pre-existing dependency	64 (37.9)
Institutionalized	14 (8.3)
Taking immunosuppressants	12 (7.1)
Taking oral corticosteroids	14 (8.3)
Hospitalization in the previous 12 months	45 (26.6)
	**Median (Q1–Q3) ^1^**
**Age**	77.1 (62.7–84.6)
**Lab Tests at Admission**	
Hematocrit (%)	35 (30–40)
Leukocytes (×10^9^/L)	10.6 (7.6–14.5)
Creatinine (mg/dL)	0.90 (0.65–1.88)
Urea (mg/dL)	56. 5 (38.0–84.5)
Albumin (g/dL)	2.6 (2.2–2.9)
Glucose (mg/dL)	117.5 (101.0–148.8)
Sodium (mmol/L)	139.0 (136.0–141.8)
Potassium (mmol/L)	4.1 (3.8–4.5)
Arterial pH	7.37 (7.34–7.41)
Arterial pCO_2_ (mmHg)	45.1 (40.2–52.3)
C-reactive protein (mg/L)	140.5 (62.0–210.3)
Procalcitonin (ng/mL)	0.49 (0.11–2.98)
SaO_2_/FiO_2_ ^2^	313.0 (243.0–360.0)
**Clinical Examination Variables at Admission**	
Temperature (°C)	37.0 (36.5–38.0)
Heart rate (bpm)	98.0 (81.5–116.8)
Respiratory rate (rpm)	20.0 (15.0–25.0)
Systolic blood pressure (mmHg)	125.0 (110.0–141.8)
Diastolic blood pressure (mmHg)	68.0 (60.0–78.0)
SatO_2_ (%) ^3^	92.0 (88.0–97.0)
Barthel Index	100 (60.0–100)
CURB 65 ^4^	2.0 (2.0–2.0)
Number of comorbidities	2.0 (1.0–3.0)

^1^ Q1: Quartile 1; Q3: Quartile 3. ^2^ SaO_2_/FiO_2_: Arterial Oxygen Saturation to Fraction of Inspired Oxygen Ratio. ^3^ SatO_2_: Oxygen saturation. ^4^ CURB 65: Community-Acquired Pneumonia Severity Index.

**Table 3:** Clinical and Laboratory Diagnosis. Complications. Health documentation.

Clinical Diagnosis	N (%)	Laboratory Diagnosis	N (%)
Pulmonary Auscultation		**Patients with Microbiological Analysis**	
Good vesicular breath sounds without pathological noises	34 (20)	Yes	160 (95.2)
Good vesicular breath sounds with pathological noises	72 (42.9)	**Sample Collection Site**	
Decreased vesicular breath sounds without pathological noises	15 (8.9)	Emergency Department	132 (78.1)
Decreased vesicular breath sounds with pathological noises	47 (28.0)	Hospital Ward	118 (69.8)
Infiltrate		Type of Sample Collected ^2^ *	
Yes	116 (98.2)	Spontaneous or induced sputum culture	126 (30.0)
Alveolar	140 (82.8)	Tracheal aspirate	6 (1.4)
Interstitial	13 (7.7)	Urinary antigen test	160 (38.1)
Mixed	13 (7.7)	Blood culture	128 (30.5)
Respiratory Support		Sample Where Pathogen Was Identified ^3^ *	
Nasal cannula	98 (58.7)	Spontaneous or induced sputum culture	167 (85.2)
Oxygen mask	22 (13.2)	Tracheal aspirate	0 (0)
HFNO ^1^	3 (1.8)	Urinary antigen test	19 (9.7)
Non-Invasive Mechanical Ventilation (NIV)	9 (5.4)	Blood culture	10 (5.1)
Invasive Mechanical Ventilation (IMV)	6 (3.6)		
No respiratory support	29 (17.4)		
**Treatments**	**N (%)**	**Complications**	**N (%)**
Use of vasopressors	7 (4.1)	Acute Respiratory Distress Syndrome (ARDS)	62 (36.7)
Nebulizers	30 (17.8)	Stroke (Cerebrovascular Accident)	1 (0.6)
Inhalers	125 (74.0)	Acute Kidney Injury (AKI)	39 (23.1)
Systemic corticosteroids	116 (68.6)	Septic Shock	18 (10.7)
**Mortality**	**N (%)**	Extrapulmonary Infection	25 (14.8)
Intra-episode death	13 (7.7)	Arrhythmias	15 (8.9)
Intra-episode and/or within 30 days post-discharge death	28 (16.6)	Congestive Heart Failure	71 (42.0)
		Myocardial Ischemia	7 (4.1)
		Altered Mental Status	32 (18.9)
		Admission to Intensive Care Unit (ICU)	9 (5.3)
**Electronic Health Record Documentation**		**N (%)**	
Suspected aspiration: dysphagia test performed		3 (1.8)	
Discharge report: secondary diagnoses included		162 (95.9)	
Therapeutic effort limitation indicated		77 (45.6)	
Prior Pneumococcal Vaccination		94 (55.6)	

^1^ HFNO: High-Flow Nasal Oxygen. ^2^ Total samples: 420. ^3^ Total samples: 196. * Note: A single patient could contribute multiple microbiological samples. Positive samples refer to sample-level identifications (n = 196), while targeted antibiotic therapy refers to patient-level pathogen identification (64/169 patients).

**Table 4:** Protocol Implementation. Stay Duration.

Proper Protocol Implementation	N (%)
** ** **N°1. Sample collection in Emergency Department**	132 (78.6)
** ** **N°2. Sample collection in Hospital Ward**	118 (70.2)
** ** **N°3. Empirical antibiotic according to guidelines**	107 (63.3)
** ** **N°4. Appropriate dose and route of empirical antibiotic according to guidelines**	104 (61.5)
** ** **N°5. Review of microbiological sample results**	161 (95.3)
** ** **N°6. Adjustment of initial empirical antibiotic to detected pathogen ^1^**	49 (77.8)
** ** **N°7. Modification of empirical treatment initiated ^2^**	65 (38.5)
Performed and guideline-compliant	48 (28.4)
Performed but not guideline-compliant	17 (10.1)
** ** **N°8. Adjustment of targeted treatment to detected pathogen ^3^**	52 (81.3)
** ** **N°9. Adequacy of targeted treatment in dose and route ^4^**	55 (84.6)
** ** **Rescue Actions (N°2 y N°5)**	
None	36 (21.3)
One rescue action	83 (49.1)
Two rescue actions	50 (29.6)
	**Median (Q1–Q3) ^5^** 1.0 (1.0–2.0) 4.0 (2.5–5.0)
** ** **Rescue Actions (N°2 y N°5)**	
** ** **Primary Actions (N° 1. 3. 4 6** **–** **9)**	
** ** **Total Actions**	5.0 (4.0–7.0)
** ** **Time to Treatment Initiation (days)**	0 (0–1)
** ** **Ward Stay Duration (days)**	5.9 (3.7–8.4)
** ** **Total Length of Stay from Emergency Admission (days)**	7.5 (4.8–9.8)

^1^ Denominator = 63 (there are 105 untested and 1 missing). ^2^ Denominator = 65 (there are 99 unmodified and 5 missing). ^3^ Denominator = 64 (there are 104 untested and 1 missing). ^4^ Denominator = 65 (there are 103 untested and 1 missing). ^5^ Q1: First quartile; Q3: Third quartile.

**Table 5:** Intra-episode mortality.

	No Mortality N (%)	Intra-Episode Mortality N (%)	Significance
**Total**	156 (93.3%)	13 (7.7%)	
**Gender Male** (N = 169)	84 (53.8)	6 (46.2)	0.593 ^a^
**Comorbidities**			
Active smoking (N = 167)	35 (22.7)	2 (15.4)	0.735 ^b^
Active alcoholism (N = 167)	28 (18.2)	2 (15.4)	1 ^b^
Substance abuse (N = 169)	8 (5.2)	0 (0)	1 ^b^
Immunocompromised (N = 167)	27 (17.3)	2 (15.4)	1 ^b^
Active neoplastic disease (N = 169)	16 (10.3)	2 (15.4)	0.633 ^b^
Chronic heart failure (N = 167)	61 (39.0)	6 (69.2)	0.038 * ^a^
Chronic liver failure (N = 169)	10 (6.4)	0 (0)	1 ^b^
Chronic kidney failure (N = 169)	31 (19.9)	2 (15.4)	1 ^b^
Previous neurological deficit (N = 169)	32 (20.5)	4 (30.8)	0.478 ^b^
Chronic obstructive pulmonary disease (COPD) (N = 169)	29 (18.6)	4 (30.8)	0.258 ^b^
Diabetes mellitus (N = 169)	45 (28.8)	1 (7.7)	0.118 ^b^
Previous dysphagia (N = 169)	7 (4.5)	1 (7.7)	0.482 ^b^
**Other potential risk factors**			
Previous dependency (N = 169)	57 (36.5)	7 (53.8)	0.243 ^b^
Institutionalized (N = 168)	11 (7.1)	3 (23.1)	0.080 ^b^
Use of immunosuppressants (N = 169)	11 (7.1)	1 (7.7)	1 ^b^
Use of oral corticosteroids (N = 169)	13 (8.3)	1 (7.7)	1 ^b^
Hospitalization in the last 12 months (N = 169)	43 (27.6)	2 (15.4)	0.517 ^b^
Infiltrate (N = 169)	156 (98.1)	13 (100)	1 ^b^
**Respiratory Support** (N = 167)			
None	29 (18.8)	0 (0)	
Conventional oxygen therapy (Nasal cannula or mask)	113 (73.4)	7 (53.8)	
HFNO ^1^	3 (1.9)	0 (0)	
Non-invasive mechanical ventilation	6 (3.9)	3 (23.1)	
Invasive mechanical ventilation	3 (1.9)	3 (23.1)	<0.001 * ^a^
**Complications During Hospitalization**			
Acute Respiratory Distress Syndrome (ARDS) (N = 169)	51 (32.7)	11 (84.6)	<0.001 * ^b^
Stroke (Cerebrovascular Accident) (N = 169)	0 (0)	1 (7.7)	0.077 ^b^
Acute Kidney Failure (N = 169)	33 (21.2)	6 (46.2)	0.078 ^b^
Septic Shock (N = 169)	15 (9.6)	3 (23.1)	0.147 ^b^
Extrapulmonary Infection (N = 169)	22 (14.1)	3 (23.1)	0.412 ^b^
Arrhythmias (N = 169)	12 (7.7)	3 (23.1)	0.094 ^b^
Congestive Heart Failure (N = 169)	62 (39.7)	9 (69.2)	0.038 * ^a^
Myocardial Ischemia (N = 168)	5 (3.2)	2 (15.4)	0.093 ^b^
Altered Mental Status (N = 169)	26 (16.7)	6 (46.2)	0.019 * ^b^
Admission to ICU ^2^ (N = 169)	6 (3.8)	3 (23.1)	0.023 * ^b^
**Treatment**			
Use of vasopressors (N = 169)	5 (3.2)	2 (15.4)	0.092 ^b^
Nebulizers (N = 169)	26 (16.7)	4 (30.8)	0.250 ^b^
Inhalers (N = 169)	114 (73.1)	11 (84.6)	0.518 ^b^
Systemic corticosteroids (N = 169)	104 (66.7)	12 (92.3)	0.065 ^b^
**Proper Protocol Implementation**			
N°1 Sample collection in Emergency Department (N = 168)	124 (80.0)	8 (61.5)	0.155 ^b^
N°2 Sample collection in Hospital Ward (N = 168)	107 (69.0)	11 (84.6)	0.348 ^b^
N°3 Empirical antibiotic according to guidelines (N = 168)	101 (65.2)	6 (46.2)	0.230 ^b^
N°4 Appropriate dose and route of empirical antibiotic according to guidelines (N = 168)	98 (63.2)	6 (46.2)	0.246 ^b^
N°5 Review of microbiological sample results (N = 168)	149 (96.1)	12 (92.3)	0.437 ^b^
N°6 Adjustment of initial empirical antibiotic to detected pathogen (N = 63)	46 (78.0)	3 (75.0)	1 ^b^
N°7 Modification of empirical treatment initiated (N = 164)	59 (39.1)	6 (46.2)	0.616 ^a^
N°8 Adjustment of targeted treatment to detected pathogen (N = 64)	49 (81.7)	3 (75.0)	0.574 ^b^
N°9 Adequacy of targeted treatment in dose and route (N = 65)	52 (85.2)	3 (75.0)	0.496 ^b^
**Rescue Actions** (N = 169)			
None	34 (21.8)	2 (15.4)	
One rescue action	78 (50.0)	5 (38.5)	
Two rescue actions	44 (28.2)	6 (46.2)	0.394 ^a^
**Electronic Health Record Documentation**			
Suspected aspiration: dysphagia test performed (N = 168)	3 (1.9)	0 (0)	1 ^b^
Discharge report: secondary diagnoses included (N = 168)	149 (96.1)	13 (100)	1 ^b^
Therapeutic effort limitation indicated (N = 168)	65 (41.9)	12 (92.3)	<0.001 * ^a^
Prior pneumococcal vaccination (N = 166)	86 (56.2)	8 (61.5)	0.710 ^a^
	**No mortality** **(Median (Q1** **–** **Q3) ^3^** **)**	**Intra-episode mortality (Median (Q1** **–** **Q3) ^3^** **)**	**Significance**
**Variables at Admission**			
Age	77.1 (61.2–84.1)	83.4 (71.1–89.1)	0.122 ^c^
CURB-65 score ^4^	2.0 (1.0–2.0)	3.0 (2.0–3.0)	0.001 * ^c^
Barthel index	100 (60.0–100)	80.0 (40.0–100)	0.127 ^c^
Number of comorbidities	2.0 (1.0–3.0)	2.0 (1.0–3.5)	0.978 ^c^
**Laboratory Analysis at Admission**			
Hematocrit	0.35 (0.30–0.40)	0.39 (0.31–0.42)	0.257 ^c^
Leukocytes	10.5 (7.4–13.8)	12.1 (8.0–17.4)	0.391 ^c^
Creatinine	0.90 (0.66–1.38)	1.11 (0.54–1.50)	0.955 ^c^
Urea	56.0 (38.0–82.0)	70.0 (40.0–134.5)	0.280 ^c^
Albumin	2.65 (2.20–2.90)	2.40 (1.70–2.65)	0.063 ^c^
Glucose	116.0 (100.0–148.0)	132.0 (100.5–147.0)	0.245 ^c^
Sodium	139.0 (136.0–141.0)	140.0 (134.0–143.5)	0.653 ^c^
Potassium	4.10 (3.80–4.50)	4.05 (3.23–4.28)	0.273 ^c^
Arterial pH	7.38 (7.34–7.41)	7.34 (7.22–7.44)	0.121 ^c^
Arterial PCO_2_	44.8 (40.2–52.0)	46.9 (37.8–73.7)	0.360 ^c^
C-reactive protein	140.0 (61.9–203.0)	168.0 (60.0–264.5)	0.748 ^c^
Procalcitonin	0.49 (0.11–2.78)	0.61 (0.29–5.62)	0.451 ^c^
**Clinical Examination Variables at Admission**			
SaO_2_/FiO_2_ ratio ^5^ (Mean [SD] ^6^)	315.8 (90.84)	151.2 (71.31)	<0.001 * ^d^
Temperature (Median (Q1–Q3) ^3^)	37.2 (36.5–38.0)	36.2 (36.0–36.7)	<0.001 * ^c^
Heart rate (bpm) (Mean (SD) ^6^)	99.8 (22.44)	99.9 (35.01)	0.992 ^d^
Respiratory rate (rpm) (Median (Q1–Q3) ^3^)	20.0 (15.0–20.0)	15.0 (15.0–27.5)	0.987 ^c^
Systolic blood pressure (mmHg) (Mean (SD) ^6^)	127.9 (25.85)	120.2 (28.72)	0.309 ^d^
Diastolic blood pressure (mmHg) (Median (Q1–Q3) ^3^)	70.0 (60.0–79.0)	63.0 (59.0–66.0)	0.059 ^c^
Oxygen saturation (%) ^7^	92.0 (88.0–94.0)	92.0 (82.5–96.0)	0.805 ^c^
**Proper Protocol Implementation**			
Primary Actions	4.0 (3.0–5.0)	3.0 (2.0–4.0)	0.122 ^c^
Total actions	5. 0 (4.0–7.0)	4.0 (3.0–6.0)	0.430 ^c^
Time to treatment initiation (days)	0 (0–1)	0 (0–1)	0.967 ^c^

* Statistical significance *p* < 0.05. ^1^ ONAF: High-Flow Nasal Oxygen Therapy. ^2^ ICU: Intensive Care Unit. ^3^ Q1: First Quartile; Q3: Third Quartile. ^4^ CURB-65: Severity Index for Community-Acquired Pneumonia. ^5^ SaO_2_/FiO_2_: Oxygen Saturation/Inspiratory Fraction of Oxygen. ^6^ SD: Standard Deviation. ^7^ SatO_2_: Oxygen Saturation. ^a^: Chi-square test; ^b^: Fisher’s test; ^c^: Mann–Whitney U test; ^d^: Student’s *t*-test for independent samples.

**Table 6:** Mortality within 30 days post-discharge.

	No Mortality N (%)	Mortality Within 30 Days N (%)	Significance
**Gender Male** (N = 156)	76 (53.9)	14 (50.0)	0.706 ^a^
**Comorbidities**			
Active smoking (N = 154)	34 (24.5)	3 (10.7)	0.110 ^a^
Active alcoholism (N = 154)	28 (20.1)	2 (7.1)	0.102 ^a^
Substance abuse (N = 154)	8 (5.8)	0 (0)	0.354 ^b^
Immunocompromised (N = 156)	23 (16.3)	6 (21.4)	0.583 ^b^
Active neoplastic disease (N = 156)	13 (9.2)	5 (17.9)	0.185 ^b^
Chronic heart failure (N = 154)	50 (36.0)	20 (71.4)	0.001 * ^a^
Chronic liver failure (N = 156)	9 (6.4)	1 (3.6)	1 ^b^
Chronic kidney failure (N = 156)	25 (17.7)	8 (28.6)	0.186 ^a^
Previous neurological deficit (N = 156)	28 (19.9)	8 (28.6)	0.304 ^a^
Chronic obstructive pulmonary disease (COPD) (N = 156)	26 (18.4)	7 (25.0)	0.424 ^a^
Diabetes mellitus (N = 156)	36 (25.5)	10 (35.7)	0.269 ^a^
Previous dysphagia (N = 155)	7 (5.0)	1 (3.6)	1 ^b^
**Other potential risk factors**			
Previous dependency (N = 156)	45 (31.9)	19 (67.9)	<0.001 * ^a^
Institutionalized (N = 155)	7 (5.0)	7 (25.0)	0.003 * ^b^
Use of immunosuppressants (N = 156)	11 (7.8)	1 (3.6)	0.693 ^b^
Use of oral corticosteroids (N = 156)	13 (9.2)	1 (3.6)	0.470 ^b^
Hospitalization in the last 12 months (N = 156)	36 (25.5)	9 (32.1)	0.470 ^a^
Infiltrate (N = 156)	138 (97.9)	28 (100)	1 ^b^
**Respiratory Support** (N = 154)			
None	29 (20.9)	0 (0)	
Conventional oxygen therapy (Nasal cannula or mask)	101 (72.7)	12 (80.0)	
HFNO ^1^	3 (2.2)	0 (0)	
Non-invasive mechanical ventilation	5 (3.6)	1 (6.7)	
Invasive mechanical ventilation	1 (0.7)	2 (13.3)	<0.001 * ^a^
**Complications During Hospitalization**			
Acute Respiratory Distress Syndrome (ARDS) (N = 156)	40 (28.4)	22 (78.6)	<0.001 * ^a^
Stroke (Cerebrovascular Accident) (N = 156)	0 (0)	1 (3.6)	0.166 ^b^
Acute Kidney Failure (N = 156)	25 (17.7)	14 (50.0)	<0.001 * ^a^
Septic Shock (N = 156)	10 (7.1)	8 (28.6)	0.003 * ^b^
Extrapulmonary Infection (N = 156)	17 (12.1)	8 (28.6)	0.038 * ^b^
Arrhythmias (N = 156)	10 (7.1)	5 (17.9)	0.078 ^b^
Congestive Heart Failure (N = 156)	52 (36.9)	19 (67.9)	0.002 * ^a^
Myocardial Ischemia (N = 155)	3 (2.1)	4 (14.3)	0.015 ^b^
Altered Mental Status (N = 156)	20 (14.2)	12 (42.9)	<0.001 * ^a^
Admission to ICU ^2^ (N = 156)	4 (2.8)	5 (17.9)	0.007 ^b^
**Treatment**			
Use of vasopressors (N = 156)	3 (2.1)	4 (14.3)	0.015 ^b^
Nebulizers (N = 156)	25 (17.7)	5 (17.9)	1 ^b^
Inhalers (N = 156)	102 (72.3)	23 (82.1)	0.280 ^a^
Systemic corticosteroids (N = 156)	94 (66.7)	22 (78.6)	0.215 ^a^
**Proper Protocol Implementation**			
N°1 Sample collection in Emergency Department (N = 155)	114 (81.4)	18 (64.3)	0.044 ^a^
N°2 Sample collection in Hospital Ward (N = 155)	95 (67.9)	23 (82.1)	0.131 ^a^
N°3 Empirical antibiotic according to guidelines (N = 155)	90 (64.3)	17 (60.7)	0.720 ^a^
N°4 Appropriate dose and route of empirical antibiotic according to guidelines (N = 155)	89 (63.6)	15 (53.6)	0.320 ^a^
N°5 Review of microbiological sample results (N = 155)	135 (96.4)	26 (92.9)	0.330 ^b^
N°6 Adjustment of initial empirical antibiotic to detected pathogen (N = 60)	43 (79.6)	6 (66.7)	0.403 ^b^
N°7 Modification of empirical treatment initiated (N = 151)	51 (37.5)	14 (50.0)	0.218 ^a^
N°8 Adjustment of targeted treatment to detected pathogen (N = 59)	45 (81.8)	7 (77.8)	0.672 ^b^
N°9 Adequacy of targeted treatment in dose and route (N = 61)	48 (85.7)	7 (77.8)	0.619 ^b^
**Rescue Actions** (N = 155)			
None	31 (22.0)	3 (20.0)	
One rescue action	74 (52.5)	4 (26.7)	
Two rescue actions	36 (25.5)	8 (53.3)	0.062 ^a^
**Electronic Health Record Documentation**			
Suspected aspiration: dysphagia test performed (N = 155)	2 (1.4)	1 (3.6)	0.423 ^b^
Discharge report: secondary diagnoses included (N = 155)	136 (97.1)	26 (92.9)	0.262 ^b^
Therapeutic effort limitation indicated (N = 155)	53 (37.9)	24 (85.7)	<0.001 * ^a^
Prior pneumococcal vaccination (N = 153)	77 (55.4)	17 (63.0)	0.468 ^a^
	**No Mortality** **(Median (Q1–Q3) ^3^** **)**	**Mortality within 30 days (Median (Q1–Q3) ^3^** **)**	**Significance**
**Variables at Admission**			
Age	74.9 (58.7–83.7)	84.0 (74.9–87.7)	0.002 * ^c^
CURB-65 score ^4^	2.0 (1.0–2.0)	2.0 (2.0–3.0)	0.003 ^c^
Barthel index	100 (70.0–100)	50.0 (40.0–95.0)	<0.001 * ^c^
Number of comorbidities	2.0 (1.0–3.0)	2.0 (1.3–3.0)	0.131 ^c^
**Laboratory Analysis at Admission**			
Hematocrit	0.35 (0.30–0.40)	0.33 (0.30–0.40)	0.630 ^c^
Leukocytes	10.21 (7.35–13.69)	11.39 (7.90–17.88)	0.289 ^c^
Creatinine	0.89 (0.65–1.29)	1.21 (0.67–1.80)	0.141 ^c^
Urea	54.0 (38.0–77.3)	86.5 (52.5–153.8)	0.001 * ^c^
Albumin	2.70 (2.28–2.93)	2.40 (2.05–2.70)	0.046 ^c^
Glucose	114.0 (99.0–142.75)	136.0 (112.8–218.3)	0.004 * ^c^
Sodium	139.0 (136.0–141.0)	140.0 (136.0–143.0)	0.137 ^c^
Potassium	4.10 (3.80–4.50)	4.10 (3.70–4.50)	0.690 ^c^
Arterial pH	7.38 (7.35–7.41)	7.34 (7.28–7.39)	0.005 * ^c^
Arterial PCO_2_	44.5 (40.1–50.8)	49.4 (40.3–56.4)	0.107 ^c^
C–reactive protein	143.5 (60.6–200.0)	116.5 (67.5–230.8)	0.666 ^c^
Procalcitonin	0.46 (0.11–3.12)	0.59 (0.19–2.10)	0.693 ^c^
**Clinical Examination Variables at Admission**			
SaO_2_/FiO_2_ ratio ^5^ (Mean [SD] ^6^)	323.2 (87.81)	201.9 (94.70)	<0.001 * ^d^
Temperature (Median (Q1–Q3) ^3^)	37.4 (36.5–38.2)	36.5 (36.0–37.0)	<0.001 * ^d^
Heart rate (bpm) (Mean (SD) ^6^)	100.1 (22.45)	98.6 (28.67)	0.757 ^d^
Respiratory rate (rpm) (Median (Q1–Q3) ^3^)	20.0 (15.0–25.0)	20.0 (15.0–25.0)	0.689 ^c^
Systolic blood pressure (mmHg) (Mean (SD) ^6^)	128.1 (26.45)	123.5 (24.20)	0.398 ^d^
Diastolic blood pressure (mmHg) (Median (Q1–Q3) ^3^)	70.0 (60.0–80.0)	65.0 (59.0–74.3)	0.209 ^c^
Oxygen saturation (%) ^7^	92.0 (88.0–94.8)	90.5 (85.0–95.8)	0.420 ^c^
**Proper Protocol Implementation**			
Primary Actions	4.0 (3.0–5.0)	4.0 (2.0–4.0)	0.146 ^c^
Total actions	5.0 (4.0–7.0)	5.0 (3.3–6.0)	0.698 ^c^
Time to treatment initiation (days)	0 (0–1)	0 (0–0)	0.712 ^c^

* Statistical significance *p* < 0.05. ^1^ ONAF: High-Flow Nasal Oxygen Therapy. ^2^ ICU: Intensive Care Unit. ^3^ Q1: First Quartile; Q3: Third Quartile. ^4^ CURB-65: Severity Index for Community-Acquired Pneumonia. ^5^ SaO_2_/FiO_2_: Oxygen Saturation/Inspiratory Fraction of Oxygen. ^6^ SD: Standard Deviation. ^7^ SatO_2_: Oxygen Saturation. ^a^: Chi-square test; ^b^: Fisher’s test; ^c^: Mann–Whitney U test; ^d^: Student’s *t*-test for independent samples.

**Table 7:** Multivariate Analysis Firth-penalized model: Intra-episode Mortality and 30-Day Post-Discharge Mortality.

**Intra-Episode Death**		
**Variables in the Equation**	**Adjusted OR ^a^** **(95% CI) ^b^**	***p*-Value**
Sex (Female)	2.69 (0.56–16.09)	0.221
Therapeutic Effort Limitation Indicated (Yes)	9.10 (1.36–121.57)	0.021 *
SaO_2_/FiO_2_ ^c^ (per unit increase)	0.98 (0.97–0.99)	<0.001 *
Empirical Antibiotic According to Guidelines (Yes)	0.33 (0.06–1.44)	0.140
Age (per year)	1.01 (0.95–1.07)	0.743
**Death within 30 Days after Discharge**		
	**Adjusted OR ^a^** **(95% CI) ^b^**	***p*-value**
ARDS (Yes) ^d^	4.29 (1.05–19.93)	0.043 *
SaO_2_/FiO_2_ ^c^ (per unit increase)	0.99 (0.98–1.00)	0.005 *
Age (per year)	1.06 (1.02–1.12)	0.005 *
Barthel Index (per point)	0.97 (0.94–0.99)	<0.001 *

* Statistical significance *p* < 0.05. ^a^ OR: Odds Ratio; ^b^ 95% CI: 95% confidence interval; ^c^ SaO_2_/FiO_2_: Oxygen Saturation/Inspiratory Fraction of Oxygen; ^d^ ARDS: Acute Respiratory Distress Syndrome.

## Data Availability

Confidentiality and anonymity of all included patients were ensured. Data management complied with the EU General Data Protection Regulation (Regulation [EU] 2016/679) and the Spanish Organic Law 3/2018 on Personal Data Protection and Digital Rights. Two separate databases were maintained: one linking patient identification numbers with personal data and another containing clinical data, both securely stored and managed in accordance with institutional data protection standards.

## Abbreviations

The following abbreviations are used in this manuscript:

AHRQ	Agency for Healthcare Research and Quality
ARDS	Acute Respiratory Distress Syndrome
CAP	Community-acquired pneumonia
HSJDA	Hospital San Juan de Dios del Aljarafe
ICU	Intensive care unit
INE	National Institute of Statistics
PSI	Pneumonia Severity Index
SaO_2_/FiO_2_	Arterial Oxygen Saturation to Fraction of Inspired Oxygen Ratio
SARS-CoV-2	Severe Acute Respiratory Syndrome Coronavirus 2
